# Replication of Sequence Information in Synthetic Oligomers

**DOI:** 10.1021/acs.accounts.0c00852

**Published:** 2021-02-06

**Authors:** Diego Núñez-Villanueva, Christopher A. Hunter

**Affiliations:** Yusuf Hamied Department of Chemistry, University of Cambridge, Lensfield Road, Cambridge CB2 1EW, U.K.

## Abstract

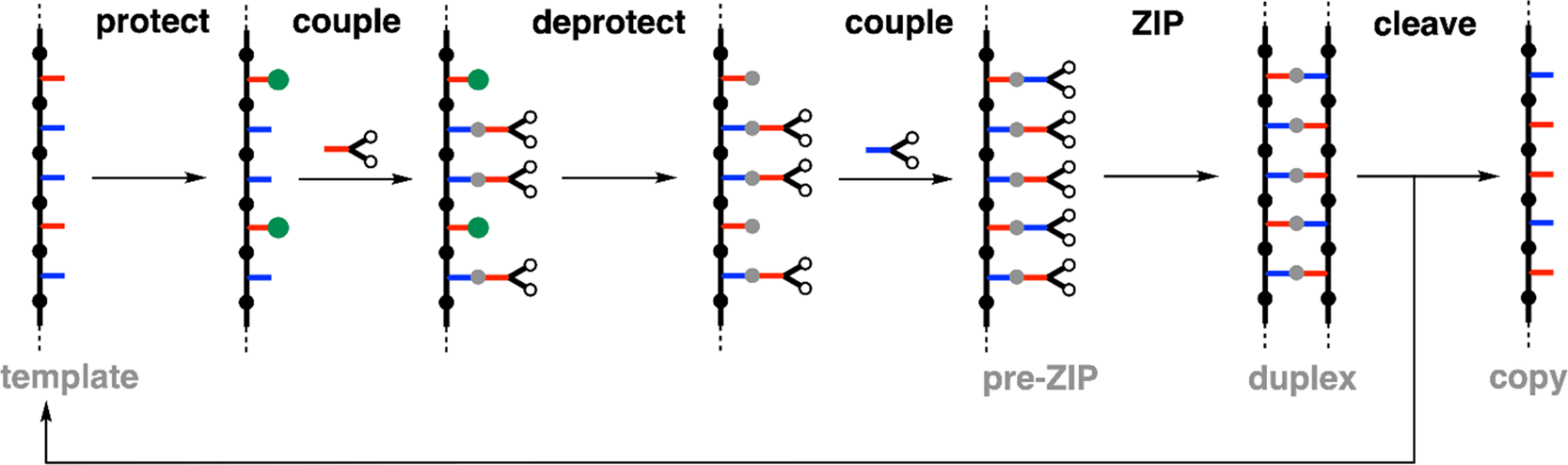

The holy grail identified by Orgel in his 1995 Account was the development of novel chemical systems that evolve using reactions in which replication and information transfer occur together. There has been some success in the adaption of nucleic acids to make artificial analogues and in templating oligomerization reactions to form synthetic homopolymers, but replication of sequence information in synthetic polymers remains a major unsolved problem. In this Account, we describe our efforts in this direction based on a covalent base-pairing strategy to transfer sequence information between a parent template and a daughter copy. Oligotriazoles, which carry information as a sequence of phenol and benzoic acid side chains, have been prepared from bifunctional monomers equipped with an azide and an alkyne. Formation of esters between phenols and benzoic acids is used as the equivalent of nucleic base pairing to covalently attach monomer building blocks to a template oligomer. Sequential protection of the phenol side chains on the template, ester coupling of the benzoic acid side chains, and deprotection and ester coupling of the phenol side chains allow quantitative selective base-pair formation on a mixed sequence template. Copper catalyzed azide alkyne cycloaddition (CuAAC) is then used to oligomerize the monomers on the template. Finally, cleavage of the ester base pairs in the product duplex by hydrolysis releases the copy strand. This covalent template-directed synthesis strategy has been successfully used to copy the information encoded in a trimer template into a sequence-complementary oligomer in high yield.

The use of covalent base pairing provides opportunities to manipulate the nature of the information transferred in the replication process. By using traceless linkers to connect the phenol and benzoic acid units, it is possible to carry out direct replication, reciprocal replication, and mutation. These preliminary results are promising, and methods have been developed to eliminate some of the side reactions that compete with the CuAAC process that zips up the duplex. *In situ* end-capping of the copy strand was found to be an effective general method for blocking intermolecular reactions between product duplexes. By selecting an appropriate concentration of an external capping agent, it is also possible to intercept macrocyclization of the reactive chain ends in the product duplex. The other side reaction observed is miscoupling of monomer units that are not attached to adjacent sites on the template, and optimization is required to eliminate these reactions. We are still some way from an evolvable synthetic polymer, but the chemical approach to molecular replication outlined here has some promise.

## Key References

- Núñez-Villanueva, D.; Ciaccia, M.; Iadevaia, G.; Sanna, E.; Hunter, C. A. Sequence Information Transfer Using Covalent Template-Directed Synthesis. Chem. Sci.2019, 10, 5258–52663119188110.1039/c9sc01460hPMC6540929.^1^*This paper describes how covalent base pairing was first implemented to make a complementary copy of a mixed sequence template*.
- Ciaccia, M.; Núñez-Villanueva, D.; Hunter C. A. Capping Strategies for Covalent Template-Directed Synthesis of Linear Oligomers Using CuAAC. J. Am. Chem. Soc.2019, 141, 10862–108753125104710.1021/jacs.9b04973.^2^*This paper describes the use of chain end-capping to control templated oligomerization reactions and introduces an experimental approach to quantifying the effective molarity for the intramolecular reactions involved in covalent templating*.
- Núñez-Villanueva, D.; Hunter C. A. Controlled Mutation in the Replication of Synthetic Oligomers. Chem. Sci.2021. DOI: 10.1039/D0SC06770APMC817950334163677.^39^*Here, manipulation of the chemistry used to connect the two components of a covalent base pair was used to change the nature of the information transferred in the copying process.*

## Introduction

Sequence information is the basis for the transmission of biological inheritance and the expression and regulation of biological function. Nucleic acids encode information as a sequence of nucleotides assembled into a linear polymeric chain. Template-directed synthesis is used to replicate this information and translate it into amino acid polymers, where the structure and function of the resulting protein are determined by sequence.^3^ The evolution of living systems, which is based on subtle variations in sequence between copy and template, is intrinsically linked to these molecular information transfer processes.^4^*In vitro* molecular evolution has been harnessed for the development of evolutionary processes to search chemical space for new functional biopolymers^5−7^ and to optimize existing biopolymers for therapeutic or manufacturing applications.^8−11^ These technologies all rely on nucleic acid replication, because no other system capable of sequence information transfer is currently known.^12,13^ Extending molecular evolution principles to synthetic polymers would allow the exploration of different regions of chemical space and the discovery of new polymer architectures where function is defined by sequence.^14^ A first step toward this goal is the development of methods for copying sequence information from one synthetic polymer to another. In this Account, we discuss the challenges, highlight some recent progress in our laboratory, and explore the prospects for molecular evolution of synthetic sequence polymers.

**Figure 1:**
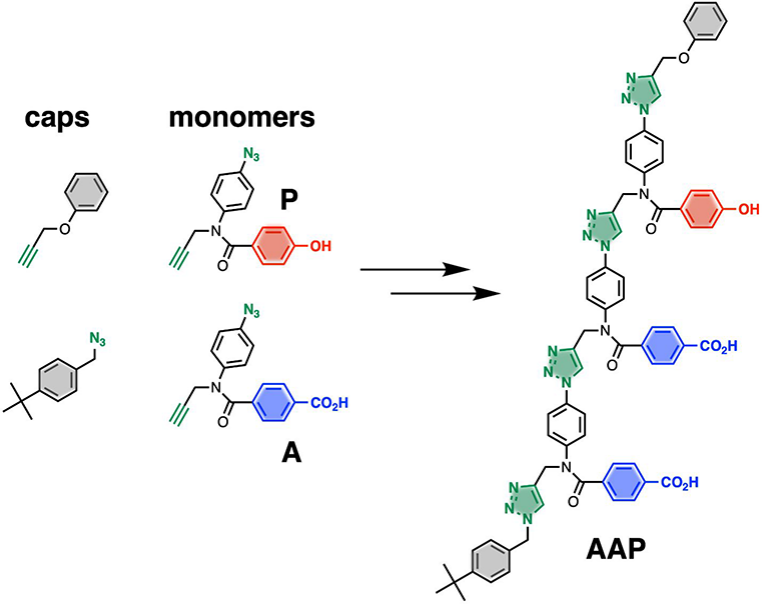
Building blocks used for the synthesis of triazole oligomers, where chemical information is encoded as the sequence of phenol (P) and benzoic acid (A) side chains. The backbone has a direction, and the sequence of an oligomer AAP is written starting from the alkyne end.

Figure 1 shows the chemical structure of the triazole oligomers that we have developed to study molecular replication. Information is encoded as a sequence of phenol and benzoic acid side chains, and copper catalyzed azide alkyne cycloaddition (CuAAC) is used for the oligomerization of azide–alkyne bifunctional monomers. The aim is to develop robust chemical methods that will allow the sequence information encoded in a template oligomer to be replicated in a copy.

## Covalent versus Noncovalent Template-Directed Synthesis

**Figure 2:**
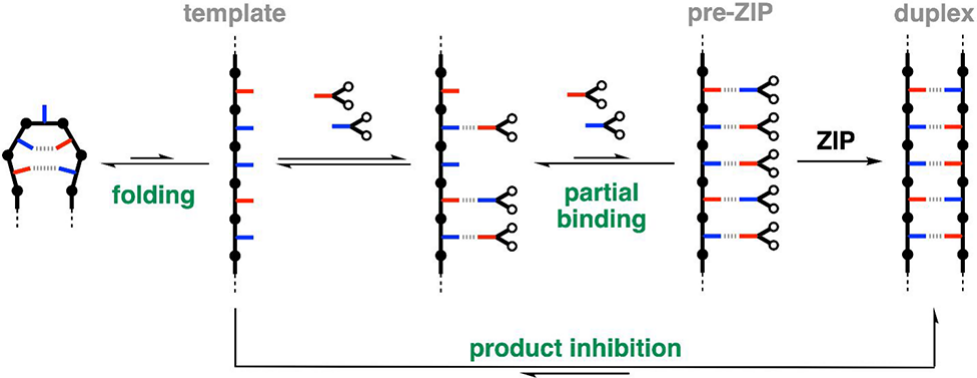
Noncovalent template-directed oligomer synthesis. The red and blue bars represent two different bases that form a base pair via noncovalent interactions represented by dashed gray lines, and the white and black dots correspond to reactive sites before and after the ZIP reaction that forms the backbone. Equilibria that compete with assembly of the key pre-ZIP intermediate are highlighted in green.

In organic synthesis, a template is used to organize an assembly of atoms in a specific spatial arrangement prior to bond formation, so that a specific product is obtained when the substrate has the potential to react in a different manner.^15^ The molecular building blocks must first be attached to the template and then removed after the reaction has taken place. Reversible chemistry is therefore required, and noncovalent templating is well-established in the field of supramolecular chemistry.^16−21^ However, Figure 2 highlights some of the difficulties in applying the noncovalent approach to the template-directed synthesis of mixed sequence oligomers. Quantitative assembly of the pre-ZIP intermediate is required to ensure highly fidelity information transfer, and there are a number of competing equilibria when the base-pairing interactions are dynamic. Incomplete binding of monomers to the template might be avoided by working at higher concentrations of monomer, but off-template intermolecular reactions will start to compete with the templated intramolecular reactions under these conditions. The availability of the template for binding to monomers is limited by intramolecular folding of mixed sequence templates. Finally, the copy will form a stable duplex with the template preventing iterative rounds of replication. In nucleic acid replication, H-bonding interactions direct the transfer of information between the template and the copy. However, the processes illustrated in Figure 2 are avoided by using enzymes to make the template accessible and control the correct attachment of monomers to the growing chain in a stepwise manner. Nonenzymatic nucleic acid replication is much less efficient.

Covalent template-directed synthesis provides an interesting solution to the problems highlighted in Figure 2. The first reports of covalent template-directed synthesis of mechanically interlocked molecules appeared in the 1960s, but with some notable exceptions, the approach has not been widely adopted.^22−34^ The use of covalent base pairs in place of the noncovalent base-pairing system found in biology opens up alternative strategies for the development of efficient sequence information transfer processes. Covalent attachment of the monomers to the template solves the problem of partial monomer binding shown in Figure 2. The ZIP step can be carried out at very high dilution to minimize any intermolecular processes that could compete with intramolecular oligomerization of the monomers on the template. The product duplex can be fully dissociated by chemical cleavage of the base pairs to recover the starting template along with the copy. Moreover, the effective molarities for intramolecular reactions where the components are held together by covalent bonds can be many orders of magnitude higher than the values observed for noncovalent systems, which would make the ZIP step very efficient.^35,36^ Another attractive feature of the covalent approach is that all of the intermediates in the process, along with any side products, can be isolated and characterized, which facilitates optimization of the chemistry.

## Kinetically Inert Base Pairs

**Figure 3:**
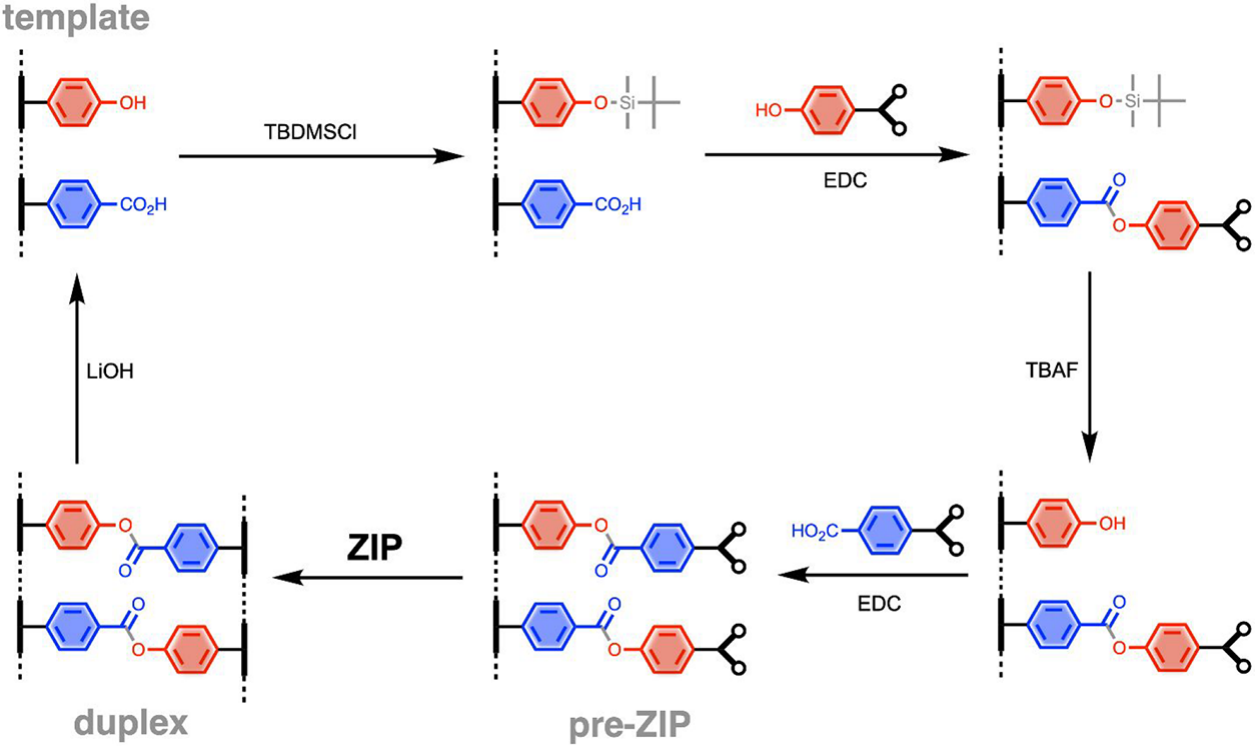
A kinetically inert base-pairing system based on ester chemistry. Selective protection followed by ester coupling attaches the phenol monomer to the template. Then deprotection followed by ester coupling step attaches the benzoic acid monomer. The base pairs are cleaved by ester hydrolysis.

The main challenge for implementing the covalent strategy is selective base-pair formation on a mixed sequence template. For polymers where information is encoded as a sequence of two complementary bases, selective protection can be used to temporarily inactivate one type of base on the template, leaving the other one available for coupling with the complementary monomer. We have developed a reliable covalent base-pairing system based on ester chemistry (Figure 3).^1^ The first step in base-pair formation is selective protection of the phenol side chains on the template, which allows selective coupling of the benzoic acid side chains with the phenol monomer. Deprotection and coupling of the phenol side chains on the template with the benzoic acid monomer gives the key pre-ZIP intermediate. This protocol prevents intramolecular covalent reactions between complementary bases on the template and solves the intramolecular folding issue encountered for noncovalent templating. Although the process used to covalently attach the monomers to the template involves multiple chemical steps, each reaction is essentially quantitative, and the products can be used in the next step without further purification after washing out excess reagents. Following the ZIP reaction, the base pairs can be cleaved by hydrolysis of the ester bonds to recover the phenol and benzoic acid side chains on the template and the copy oligomer. The irreversibility of the duplex cleavage reaction ensures that there is no possibility of product inhibition in multiple rounds of a replication cycle.

These kinetically inert covalent base pairs have distinctly different properties from dynamic covalent base pairs, because the chemical steps used for attachment and cleavage are not under equilibrium control, which is an essential requirement if the competing processes highlighted in Figure 2 are to be avoided. The use of a single type of ester base pair provides a two-letter alphabet that encodes chemical information in binary form. Additional base pairs could be used to expand the size of the alphabet and increase the density of information encoded in synthetic sequence polymers, but each new base pair requires the development of protection and coupling chemistry that is orthogonal to the chemistry used for all of the other base pairs.

## Linear versus Cyclic Templates

**Figure 4:**
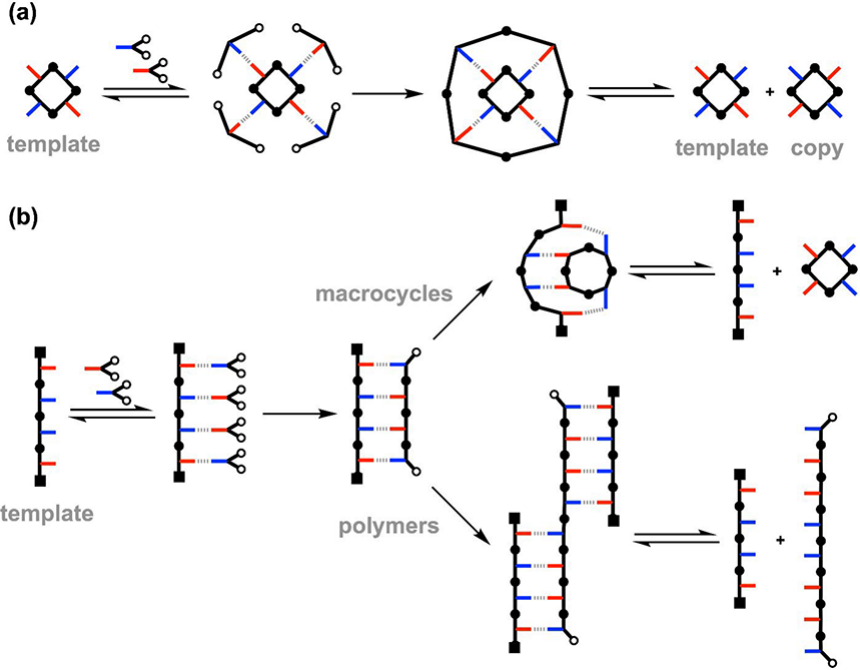
(a) In cyclic templating, the product duplex is stable, because all of the reactive sites (white dots) are consumed. (b) In linear templating, the product duplex has reactive end groups that can react further to form macrocycles or polymers.

Since the discovery of crown ethers half a century ago, template-directed synthesis has mainly been used to direct ring closure reactions for the formation of macrocycles, cages, catenanes, rotaxanes, and knots.^16−21^ An advantage of cyclic templating is that no further reactions are possible after ring closure takes place (Figure 4a). The major challenge for the development of linear templating methods is that after the intramolecular oligomerization reaction takes place on the template, the product strand still carries reactive groups on the chain ends. Further reaction of these chain ends will lead to macrocyclic and polymeric side products (Figure 4b). In Nature, start and stop sites are programmed into the nucleic acid template to ensure that the desired linear product is obtained. In order to implement linear templating in synthetic oligomers, chemical strategies are required to provide a stop signal and suppress side reactions, and one solution is described below.

## Chain End Capping

**Figure 5:**
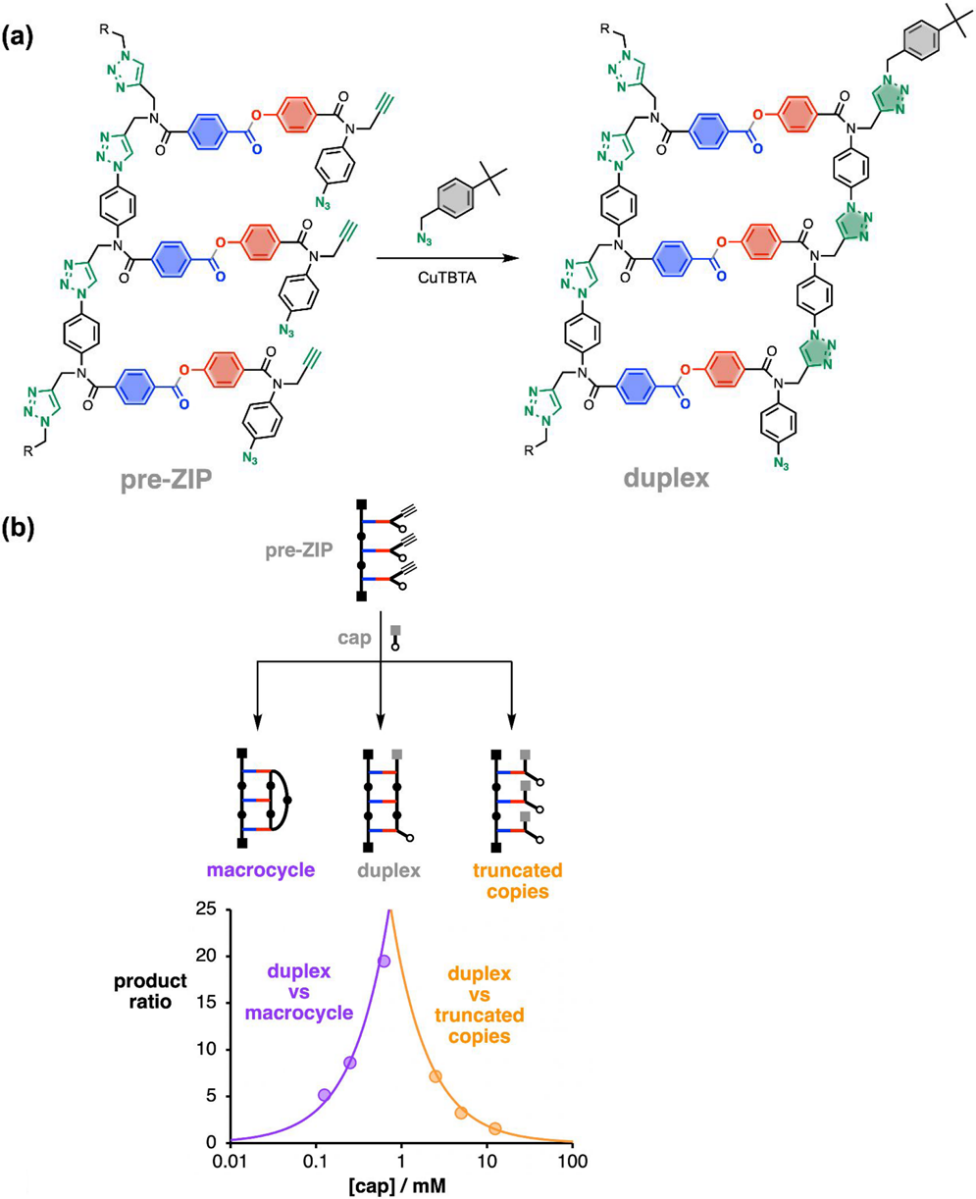
*In situ* capping of chain ends in the ZIP step. (a) When the CuAAC reaction is carried out on the pre-ZIP intermediate in the presence of an excess of 4-*tert*-butylbenzyl azide, a single major product is obtained. An antiparallel arrangement of the backbones in the duplex is shown, but the parallel product is also possible (see below). R = 3,5-di-*tert*-butylphenyl. (b) The effect of the concentration of the capping agent on the product distribution. The lines are the theoretical relationships obtained if the product ratio is directly proportional to the ratio of the concentration of the capping agent and the effective molarity for the intramolecular reaction leading to macrocylization (purple) or to zipping up the duplex (orange).^37^

One approach to minimizing the intermolecular reactions that lead to polymers is to work at high dilution, but this strategy does not affect the intramolecular side reactions that lead to macrocycles. In addition, the precise reaction mechanism is important. For the CuAAC reaction used to oligomerize the building blocks shown in Figure 1, the rate limiting step is formation of an activated copper–alkyne complex, which then reacts rapidly with the nearest available azide. Dilution therefore has little impact on the product distribution. However, addition of an azide capping agent to the oligomerization reaction provides an effective solution. Figure 5 shows that when the ZIP step is carried out in the presence of an excess of an external capping agent (4-*tert*-butylbenzyl azide), it is possible to control the oligomerization reaction to obtain a single major product. The effective molarity (EM) for the intramolecular CuAAC reaction that leads to zipping up of the duplex is about 500 mM, and the EM for intramolecular cyclization of product duplex is about 100 μM.^2,37^ By using a concentration of the capping agent in the middle of these two EM values (1 mM), it is possible to intercept the macrocylization reaction without truncating the copy obtained in the ZIP process (Figure 5b). Provided the concentration of the pre-ZIP intermediate used in the CuAAC reaction is sufficiently low (typically 25 μM), there is no possibility of intermolecular polymerization reactions competing with capping of the terminal alkyne by the external azide, which is present in a large excess.

*In situ* capping of the chain ends provides a general solution to blocking intermolecular polymerization reactions in linear templating. The criteria for success are set by the value of EM for the ZIP process. For 99% efficiency, the concentration of the capping agent should be 2 orders of magnitude lower than EM, and the concentration of pre-ZIP intermediate should be another 2 orders of magnitude lower. Therefore, by operating at sufficiently high dilution and choosing an appropriate excess of the capping agent, it should always be possible to block polymerization without interfering with the ZIP process. However, the use of capping agents to block intramolecular macrocyclization reactions is not so straightforward, because success depends on the conformational properties of the backbone. Capping agents will only be useful for intercepting macrocycle formation, if the EM for macrocyclization is orders of magnitude lower than the EM for the ZIP process. For the oligotriazole backbone shown in Figure 1, the backbone is sufficiently rigid to prevent formation of dimeric macrocycles, and the EM for cyclization of the trimer on the template is more than 3 orders of magnitude lower than the EM for the ZIP process. Rational design of these features into new oligomer architectures represents a challenge, but the supramolecular organization afforded by base stacking in nucleic acids suggests one possible strategy for controlling conformation.

## Backbone Directionality

**Figure 6:**
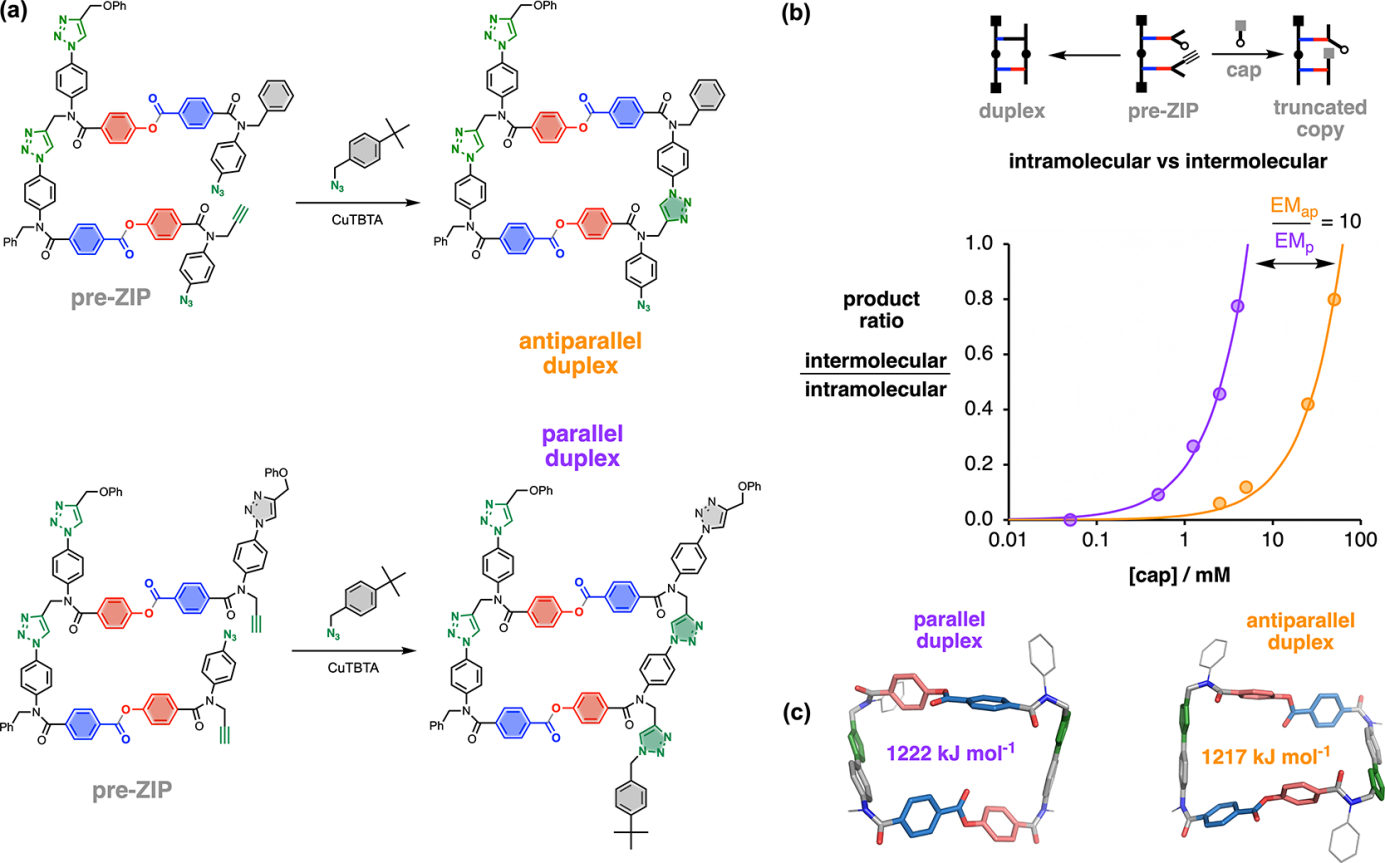
Precapped monomers determine the backbone direction of the product duplex. (a) The only possible product of the CuAAC reaction is (a) the antiparallel duplex when a terminal alkyne group is removed in the pre-ZIP intermediate or the parallel duplex when a terminal azide group is capped in the pre-ZIP intermediate. (b) Addition of increasing amounts of an external capping agent (4-*tert*-butylbenzyl azide) was used to determine values of EM through competition with the intramolecular reaction. Ten times more of the capping agent was required to compete with formation of the antiparallel duplex (orange data) than the parallel duplex (purple data). The lines are the theoretical relationships obtained if the product ratio is directly proportional to the ratio of the concentration of the capping agent and the effective molarity for the intramolecular reaction. (c) Molecular mechanics models of isomeric parallel and antiparallel duplexes suggest that the antiparallel backbone arrangement is lower in energy (MMFFs force field with chloroform solvation).^2^

There are two isomeric forms of the duplex product shown in Figure 5, because the backbone has a direction. Just as in nucleic acids, parallel and antiparallel arrangements of the two backbones are possible for the triazole oligomers, and there are important consequences for the ZIP process. By assembling pre-ZIP intermediates where one of the terminal monomers was precapped to remove either the azide or the alkyne functionality (Figure 6), it was possible to directly study CuAAC reactions in which only one of the parallel or antiparallel duplexes can be formed.^2^ Titration of 4-*tert*-butylbenzyl azide into the reaction mixtures was used to determine values of EM for the intramolecular reactions that zip up the duplex, based on the concentration of the external capping agent required to compete with the intramolecular process (Figure 6b). The EM for formation of the antiparallel duplex is an order of magnitude higher than for formation of the parallel duplex. This result is consistent with molecular mechanics calculations, which suggest that the antiparallel duplex is 5 kJ mol^–1^ more stable than the parallel isomer (Figure 6c). In experiments using uncapped monomers on the same heterodimer template, it was possible to find concentrations of the capping agent where formation of the parallel duplex was completely suppressed, giving the antiparallel duplex as the only major product.

It would be possible to avoid the issue of backbone directionality by using symmetric monomer building blocks. Otherwise controlling the exclusive formation of either parallel or antiparallel linkages in the ZIP process will be critical to the success of templating longer oligomers. This selectivity is determined by the conformational properties of the backbone and represents a significant challenge for the rational design of new oligomer architectures.

## Sequence Information Transfer

**Figure 7:**
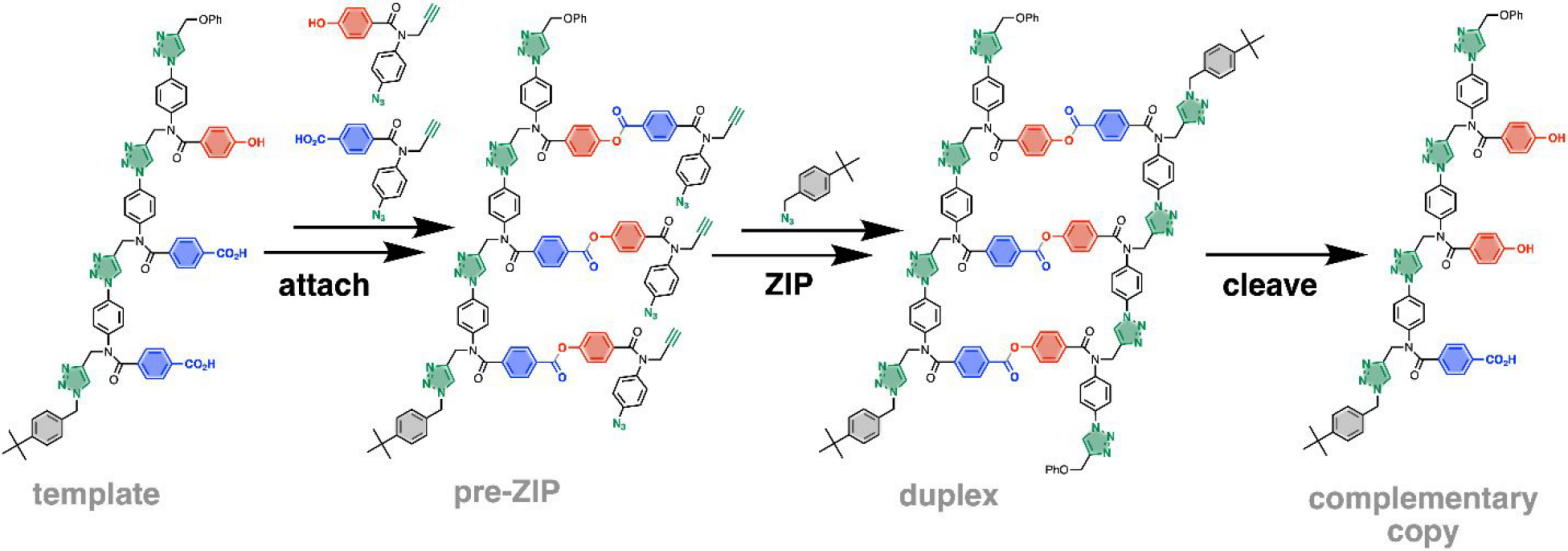
Sequence information transfer using covalent template-directed synthesis based on ester base-pair chemistry. The monomers were attached to the template (AAP) to give the pre-ZIP intermediate using the reaction sequence shown in Figure 4. A CuAAC reaction in the presence of a capping azide gave the corresponding duplex (only the major isomer is shown). The ester base pairs were cleaved by hydrolysis to regenerate the template, and capping of the terminal azide in the templated product gave the sequence-complementary copy, APP, as the major product.^1^

The combination of ester base pairing and cap-controlled backbone oligomerization provides high-yielding chemistry suitable for copying mixed sequence templates using covalent template-directed synthesis. Figure 7 illustrates the sequence information transfer process, which was carried out using a trimer template, AAP (we write the sequence starting from the alkyne terminus, A = benzoic acid and P = phenol).^1^ The two different types of monomer were loaded onto the template using the protection-coupling-deprotection-coupling reaction sequence shown in Figure 3. Base-pair formation proceeded quantitatively, and the resulting pre-ZIP intermediate was subjected to CuAAC oligomerization in the presence of a 100-fold excess of a capping azide. Cleavage of the ester base pairs gave the template and copy strands, which were separated by chromatography, and the terminal azide groups were then capped.

This seven-step cycle from template to copy proceeded with almost quantitative conversion in each step. However, spectroscopic examination of the copy revealed that it was actually a mixture of three different sequences, PPA, PAP, and APP. As yet, we have not found a reliable method for sequencing these oligomers, so identification was based on direct synthesis of the three isomeric products and comparison of the ^1^H NMR spectra. The major product (72%) corresponds to the sequence-complementary copy of the template resulting from the antiparallel duplex shown in Figure 7 (APP). The sequence-complementary copy that comes from the parallel duplex (PPA) was the minor product (11%). The remaining 17% was the scrambled sequence PAP, which comes from the intramolecular coupling between the two terminal monomer units on the template. Although we draw the backbone in an extended conformation in Figure 7, it is clear that there is sufficient flexibility for reaction between the monomers in positions 1 and 3 on the chain to compete with the desired 1,2-coupling. This long-range miscoupling is the process that limits the fidelity of sequence information transfer in this system, but reducing the probability of miscoupling requires changes in the conformational properties of the backbone.

## Reprogramming the Information Transfer Process

**Figure 8:**
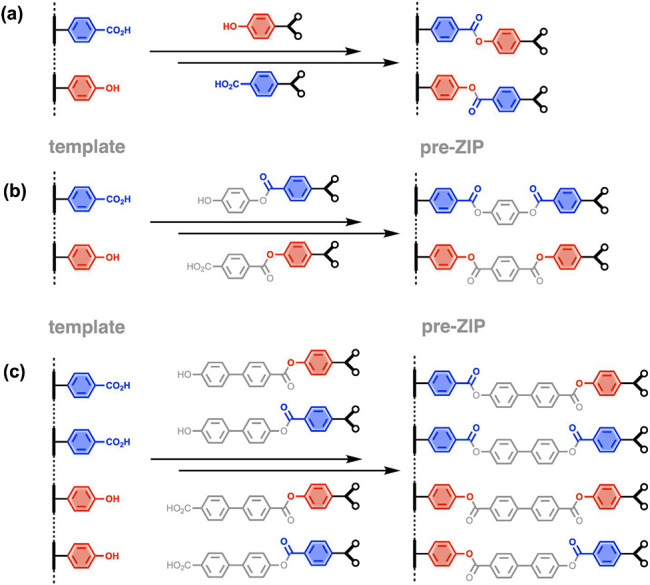
Information transferred from the parent to the daughter strand can be programmed by using base pairs connected by traceless linkers (gray). (a) Reciprocal replication. A base pair formed by the direct attachment of benzoic acid and phenol units results in a complementary copy of the template. (b) Direct replication. Connecting two identical bases via a symmetric linker results in an identical copy of the template after cleavage of all of the ester bonds to release the linker. (c) Replication with mutation. Isosteric linkers can be used to introduce mixtures of symmetric and unsymmetric base pairs, which result in simultaneous direct and reciprocal copying.

The use of kinetically inert covalent base pairing opens interesting new opportunities that are not so accessible with noncovalent or dynamic approaches. We have investigated the use of traceless linkers to reprogram the nature of the information transferred in the copying process (Figure 8). The phenol–benzoic acid base-pairing system described above leads to reciprocal copying of chemical information, analogous to nucleic acid replication (Figure 8a). It is possible to use the same ester chemistry to achieve direct replication by incorporating traceless linkers to connect two identical components in symmetrical base pairs. Figure 8b shows how a hydroquinone linker can be used to connect two benzoic acids, and a terephthalic acid linker can be used to connect two phenols. If the monomer building blocks shown in Figure 7 were equipped with these linkers, then the replication cycle shown in Figure 7 would result in a copy that has the same sequence as the starting template. The linkers are removed in the cleave step, because the two ester bonds at each end of the linker will be hydrolyzed. We have demonstrated the viability of this approach by carrying out iterative rounds of replication using AAA as a template and benzoic acid monomers equipped with a hydroquinone linker.^38^

## Controlled Mutation

**Figure 9:**
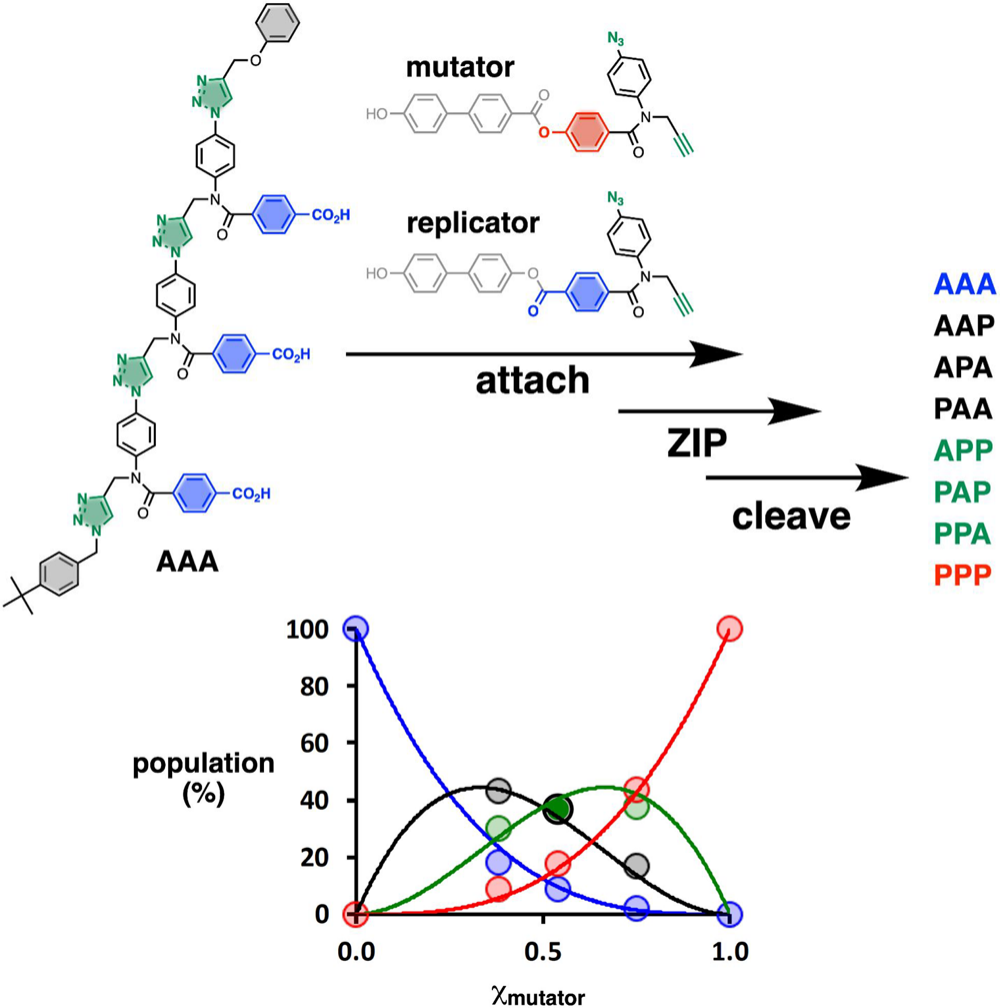
Product distributions for covalent template-directed replication of an AAA template in the presence of different amounts of a mutator monomer (χ_mutator_). The population of the direct copy AAA is shown in blue, products with a single phenol mutation are shown in black, two phenol mutations are shown in green, and the fully mutated reciprocal copy PPP is shown in red. Calculated statistical distributions are shown as lines, and the experimental results are shown as dots.^39^

The development of covalent template-directed replication of synthetic oligomers represents a first step toward the application of directed evolution to non-natural oligomers. However, searching sequence space using molecular evolution requires a process where each round of replication generates a new population of copy strands, which are different from the parent population. In other words, we require replication with mutation. The rate of mutation must be high enough to introduce a significant population of new sequences but low enough to make sure the information contained in the parent population is not lost. The traceless linker base-pairing scheme in Figure 8 provides an ideal method not only for introducing mutations into the replication process but also for precisely controlling the mutation rate. Figure 8c shows a set of isosteric base pairs, which could be used interchangeably within the same duplex. The symmetrical base pairs lead to direct replication of the sequence information in the template, and the unsymmetrical base pairs lead to reciprocal replication. Thus, by spiking the symmetrical base pairs with small amounts of the unsymmetrical base pairs, it should be possible to introduce point mutations at a rate directly determined by the proportions of the different monomers used in the attach step. We have demonstrated the viability of this approach by copying AAA in the presence of different proportions of a monomer that leads to replication, i.e., copying acid to acid, and a monomer that leads to mutation, i.e., copying acid to phenol (Figure 9).^39^ The population of different sequences present in the product mixture obtained after the seven-step replication cycle can be accurately predicted based on statistical incorporation of the mutator monomer.

## Conclusions and Future Perspectives

The development of synthetic polymers in the 20th century has transformed the way we live. Function is generally related to bulk material properties, and the structures are generally homopolymers or block copolymers. In contrast, biology uses copolymers where the detailed sequence of monomers is used to achieve a much broader range of functional properties. It seems likely that function could be programmed into synthetic polymers using a sequence of different monomer building blocks in the same way as biopolymers. However, there are two significant challenges to the development of the chemistry of synthetic sequence polymers: the synthesis of long chains of defined sequence is a practical challenge, and the only viable method currently available is solid phase synthesis, which is scale limited; and as chain length increases, there is a combinatorial explosion in the number of possible sequences, so finding out which ones have interesting properties and are worth making is an even more difficult challenge. The biological solution to both of these problems is based on template synthesis. We have therefore begun to investigate approaches to templating the sequence of synthetic polymers. Our preliminary results based on covalent templated-directed synthesis are summarized here.

Inspired by the H-bonded base pairs found in nucleic acids, there has been some success in the development of chemical replication systems based on the use of dynamic interactions to template the coupling of two monofunctional building blocks. Oligomerization of bifunctional monomers is more challenging, because there are multiple competing equilibria and reaction pathways. The use of kinetically inert covalent bonds to attach monomers to a template offers one possible solution. We have developed covalent base-pairing chemistry based on formation of an ester between a phenol and a benzoic acid, which can be used to quantitatively attach two different monomer building blocks to a mixed sequence template. Each monomer is equipped with an alkyne and an azide, and high yielding oligomerization can be achieved using CuAAC reactions to give a covalently linked duplex. Subsequent cleavage of the base pairs by hydrolysis regenerates the template and a copy strand, which can be used in another round of replication. It is possible to manipulate the nature of the information which is transferred in this replication process by changing the chemical structure of the base pair. We have used traceless linkers to achieve direct replication and reciprocal replication and to introduce mutations, where the error rate can be precisely controlled.

**Figure 10:**
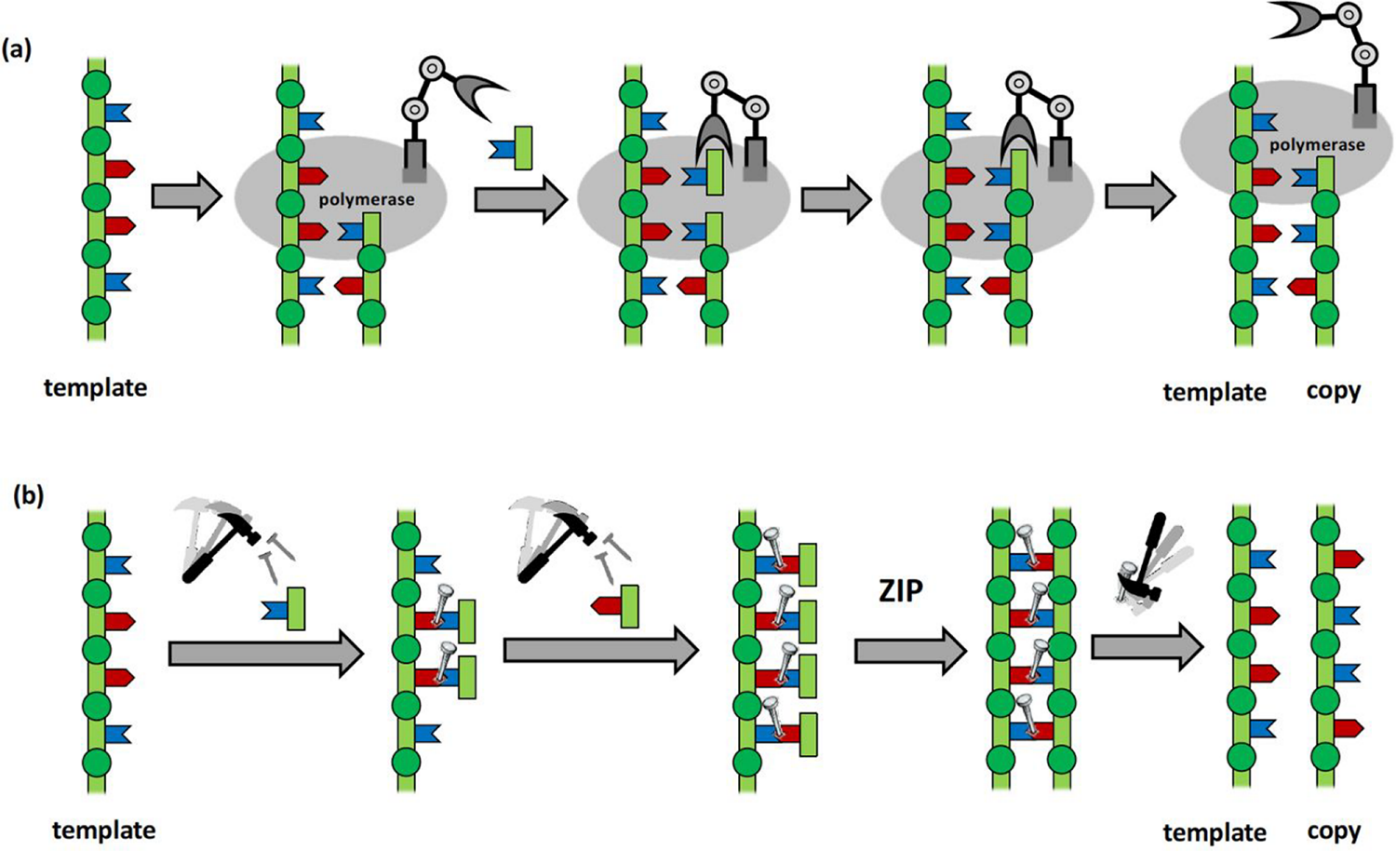
(a) Stepwise information transfer using kinetically labile base pairs in nucleic acids controlled by a polymerase. (b) Parallel information transfer using kinetically inert base pairs is an alternative chemical approach.

Figure 10 compares the chemical process we have developed with replication of sequence information in biology. In nucleic acid replication, a polymerase progressively adds monomer units stepwise onto a growing copy strand, and the fidelity of sequence information transfer is determined by how well each H-bonded base pair fits into a binding pocket in the protein. In covalent template-directed synthesis, the monomers are all attached to the template first, and the fidelity of this process is limited by chemical yield. Oligomerization of all of the monomer units then takes place in parallel in a single reaction step. Although the chemical replication cycle involves a total of seven reaction steps, provided each step is sufficiently high yielding, this approach should scale well, because the number of steps required is independent of the length of the template. The real challenge for development of a robust chemical replication process using longer oligomers is controlling the conformational properties of the backbone. The major side reactions that we have identified occur in the ZIP step: macrocyclization of chain ends of the copy strand attached to the template and miscoupling between two monomers that are not attached to adjacent sites on the template. Both processes are related to conformational flexibility in the pre-ZIP intermediate. Taking inspiration from nucleic acids would suggest that a combination of a relatively rigid backbone and supramolecular self-organization should provide promising strategies for future exploration.

## References

[ref1] Núñez-Villanueva D.; Ciaccia M.; Iadevaia G.; Sanna E.; Hunter C. A. Sequence Information Transfer Using Covalent Template-Directed Synthesis. Chem. Sci. 2019, 10, 5258–5266. 10.1039/C9SC01460H. 31191881 PMC6540929

[ref2] Ciaccia M.; Núñez-Villanueva D.; Hunter C. A. Capping Strategies for Covalent Template-Directed Synthesis of Linear Oligomers Using CuAAC. J. Am. Chem. Soc. 2019, 141, 10862–10875. 10.1021/jacs.9b04973. 31251047

[ref39] Núñez-Villanueva D.; Hunter C. A. Controlled Mutation in the Replication of Synthetic Oligomers. Chem. Sci. 2021, 10.1039/D0SC06770A. PMC8179503 34163677

[ref3] Sinden R. R. DNA Structure and Function; Academic Press: 1994; 10.1016/C2009-0-02451-9.

[ref4] Griffiths A. J. F.; Gelbart W. M.; Miller J. H.; Lewontin R. C. Modern genetic analysis; Freeman: 1999.

[ref5] Tuerk C.; Gold L. Systematic Evolution of Ligands by Exponential Enrichment: RNA Ligands to Bacteriophage T4 DNA Polymerase. Science 1990, 249, 505–510. 10.1126/science.2200121. 2200121

[ref6] Ellington A. D.; Szostak J. W. *In Vitro* Selection of RNA Molecules That Bind Specific Ligands. Nature 1990, 346, 818–822. 10.1038/346818a0. 1697402

[ref7] Joyce G. F. Forty Years of *In Vitro* Evolution. Angew. Chem., Int. Ed. 2007, 46, 6420–6436. 10.1002/anie.200701369. 17634987

[ref8] Chen K.; Arnold F. H. Tuning the Activity of an Enzyme for Unusual Environments: Sequential Random Mutagenesis of Subtilisin E for Catalysis in Dimethylformamide. Proc. Natl. Acad. Sci. U. S. A. 1993, 90, 5618–5622. 10.1073/pnas.90.12.5618. 8516309 PMC46772

[ref9] Famulok M.; Mayer G.; Blind M. Nucleic Acid Aptamers - From Selection in Vitro to Applications in Vivo. Acc. Chem. Res. 2000, 33, 591–599. 10.1021/ar960167q. 10995196

[ref10] Turner N. J. Directed evolution drives the next generation of biocatalysts. Nat. Chem. Biol. 2009, 5, 567–573. 10.1038/nchembio.203. 19620998

[ref11] Bornscheuer U. T.; Hauer B.; Jaeger K. E.; Schwaneberg U. Directed Evolution Empowered Redesign of Natural Proteins for the Sustainable Production of Chemicals and Pharmaceuticals. Angew. Chem., Int. Ed. 2019, 58, 36–40. 10.1002/anie.201812717. 30520553

[ref12] Böhler C.; Nielsen P. E.; Orgel L. E. Template Switching Between PNA and RNA Oligonucleotides. Nature 1995, 376, 578–581. 10.1038/376578a0. 7543656

[ref13] Brudno Y.; Liu D. R. Recent Progress Toward the Templated Synthesis and Directed Evolution of Sequence-Defined Synthetic Polymers. Chem. Biol. 2009, 16, 265–276. 10.1016/j.chembiol.2009.02.004. 19318208 PMC2692969

[ref14] Orgel L. E. Unnatural Selection in Chemical Systems. Acc. Chem. Res. 1995, 28, 109–118. 10.1021/ar00051a004. 11542502

[ref15] Anderson S.; Anderson H. Templates in Organic Synthesis: Definitions and Roles. In Templated Organic Synthesis; Stang P., Diederich F., Eds.; Wiley-VCH Verlag GmbH: Weinheim, 2000; pp 1–38, 10.1002/9783527613526.ch01.

[ref16] Pedersen C. J. Cyclic Polyethers and Their Complexes with Metal Salts. J. Am. Chem. Soc. 1967, 89, 7017–7036. 10.1021/ja01002a035.

[ref17] Dietrich-Buchecker C. O.; Sauvage J. P.; Kintzinger J. P. Une nouvelle famille de molecules: les metallo-catenanes. Tetrahedron Lett. 1983, 24, 5095–5098. 10.1016/S0040-4039(00)94050-4.

[ref18] Hunter C. A. Synthesis and Structure Elucidation of a New [2]-Catenane. J. Am. Chem. Soc. 1992, 114, 5303–5311. 10.1021/ja00039a047.

[ref19] Anderson S.; Anderson H. L.; Sanders J. K. M. Expanding roles for templates in synthesis. Acc. Chem. Res. 1993, 26, 469–475. 10.1021/ar00033a003.

[ref20] Ashton P. R.; Goodnow T. T.; Kaifer A. E.; Reddington M. V.; Slawin M. Z.; Spencer N.; Stoddart J. F.; Vincent C.; Williams D. J. A [2] Catenane Made to Order. Angew. Chem., Int. Ed. Engl. 1989, 28, 1396–1399. 10.1002/anie.198913961.

[ref21] Ayme J.-F.; Beves J. E.; Campbell C. J.; Leigh D. A. Template Synthesis of Molecular Knots. Chem. Soc. Rev. 2013, 42, 1700–1712. 10.1039/C2CS35229J. 22890502

[ref22] Schill G.; Luttringhaus A. The Preparation of Catena Compounds by Directed Synthesis. Angew. Chem., Int. Ed. Engl. 1964, 3, 546–547. 10.1002/anie.196405461.

[ref23] Moneta W.; Baret P.; Pierre J. Design and Syntheses of Macrocyclic Hosts containing Convergent Hydroxy Groups. J. Chem. Soc., Chem. Commun. 1985, 899–901. 10.1039/c39850000899.

[ref24] Höger S.; Meckenstock A.-D.; Pellen H. High-Yield Macrocyclization via Glaser Coupling of Temporary Covalent Templated Bisacetylenes. J. Org. Chem. 1997, 62, 4556–4557. 10.1021/jo970350c.

[ref25] Höger S.; Meckenstock A.-D. Template-Directed Synthesis of Shape-Persistent Macrocyclic Amphiphiles with Convergently Arranged Functionalities. Chem. - Eur. J. 1999, 5, 1686–1691. 10.1002/(SICI)1521-3765(19990604)5:6<1686::AID-CHEM1686>3.0.CO;2-0.

[ref26] Hiratani K.; Kaneyama M.; Nagawa Y.; Koyama E.; Kanesato M. Synthesis of [1]Rotaxane via Covalent Bond Formation and its Unique Fluorescent Response by Energy Transfer in the Presence of Lithium Ion. J. Am. Chem. Soc. 2004, 126, 13568–13569. 10.1021/ja046929r. 15493885

[ref27] Kawai H.; Umehara T.; Fujiwara K.; Tsuji T.; Suzuki T. Dynamic Covalently Bonded Rotaxanes Cross-Linked by Imine Bonds between the Axle and Ring: Inverse Temperature Dependence of Subunit Mobility. Angew. Chem., Int. Ed. 2006, 45, 4281–4286. 10.1002/anie.200600750. 16739149

[ref28] Schweez C.; Shushkov P.; Grimme S.; Höger S. Synthesis and Dynamics of Nanosized Phenylene-Ethynylene-Butadiynylene Rotaxanes and the Role of Shape Persistence. Angew. Chem., Int. Ed. 2016, 55, 3328–3333. 10.1002/anie.201509702. PMC4797704 26836984

[ref29] Steemers L.; Wanner M. J.; Lutz M.; Hiemstra H. Van Maarseveen, J. H. Synthesis of Spiro Quasi[1]Catenanes and Quasi[1]Rotaxanes via a Templated Backfolding Strategy. Nat. Commun. 2017, 8, 15392 10.1038/ncomms15392. 28541349 PMC5458513

[ref30] Zimmerman S. C.; Wendland M. S.; Rakow N. A.; Zharov I.; Suslick K. S. Synthetic Hosts by Monomolecular Imprinting Inside Dendrimers. Nature 2002, 418, 399–403. 10.1038/nature00877. 12140553

[ref31] Zimmerman S. C.; Zharov I.; Wendland M. S.; Rakow N. A.; Suslick K. S. Molecular Imprinting Inside Dendrimers. J. Am. Chem. Soc. 2003, 125, 13504–13518. 10.1021/ja0357240. 14583047

[ref32] Lin N.-T.; Lin S.-Y.; Lee S.-L.; Chen C.-h.; Hsu C.-H.; Hwang L.-P.; Xie Z.-Y.; Chen C.-H.; Huang S.-L.; Luh T.-Y. From Polynorbornene to the Complementary Polynorbornene by Replication. Angew. Chem., Int. Ed. 2007, 46, 4481–4485. 10.1002/anie.200700472. 17455179

[ref33] Ke Y.-Z.; Lee S.-L.; Chen C.-h.; Luh T.-Y. Unsymmetrical Polymeric Ladderphanes by Sequential Polymerization: a New Approach for the Template Synthesis of Polymers with Well-Defined Chain Length and Narrow Polydispersity. Chem. - Asian J. 2011, 6, 1748–1751. 10.1002/asia.201000877. 21341375

[ref34] Ke Y.-Z.; Ji R.-J.; Wei T.-C.; Lee S.-L.; Huang S.-L.; Huang M.-J.; Chen C.-h.; Luh T.-Y. Well-Defined Condensation Polymers with Narrow Polydispersity via Unsymmetrical Ladderphanes by Sequential Polymerization. Macromolecules 2013, 46, 6712–6722. 10.1021/ma4012363.

[ref35] Kirby A. J. Effective Molarities for Intramolecular Reactions. Adv. Phys. Org. Chem. 1980, 17, 183–278. 10.1016/S0065-3160(08)60129-X.

[ref36] Motloch P.; Hunter C. A. Thermodynamic Effective Molarities for Supramolecular Complexes. Adv. Phys. Org. Chem. 2016, 50, 77–118. 10.1016/bs.apoc.2016.07.001.

[ref37] Núñez-Villanueva D.; Ciaccia M.; Hunter C. A. Cap Control: Cyclic Versus Linear Oligomerisation in Covalent Template-Directed Synthesis. RSC Adv. 2019, 9, 29566–29569. 10.1039/C9RA07233K. PMC9071899 35531529

[ref38] Núñez-Villanueva D.; Hunter C. A. Molecular Replication Using Covalent Base-Pairs with Traceless Linkers. Org. Biomol. Chem. 2019, 17, 9660–9665. 10.1039/C9OB02336D. 31691702

